# Dapagliflozin-Associated Euglycemic Diabetic Ketoacidosis Presenting With Severe Abdominal Pain Mimicking Acute Peritonitis

**DOI:** 10.7759/cureus.22229

**Published:** 2022-02-15

**Authors:** Qianwen Wang, Kangze Wu, Xiaoqian Luo, Xin Dong, Weifeng Liu, Zhe Tang, Bo Zhang

**Affiliations:** 1 Department of Surgery, Fourth Affiliated Hospital, School of Medicine, Zhejiang University, Hangzhou, CHN; 2 Department of Surgery, Second Affiliated Hospital, School of Medicine, Zhejiang University, Hangzhou, CHN; 3 Department of Intensive Care Medicine, Second Affiliated Hospital, School of Medicine, Zhejiang University, Hangzhou, CHN; 4 Department of Hepatopancreatobiliary Surgery, Second Affiliated Hospital, School of Medicine, Zhejiang University, Hangzhou, CHN

**Keywords:** sugery, abdominal pain, sodium-glucose co-transporter-2 inhibitor, euglycemic diabetic ketoacidosis, dapagliflozin

## Abstract

Euglycemic diabetic keto acidosis (eu-DKA) is a rare but life-threatening metabolic complication associated with sodium-glucose cotransporter-2 (SGLT-2) inhibitor therapy, especially in the setting of extreme physical stress. Due to no apparent hyperglycemia, the significant risk of delayed diagnosis or misdiagnosis exists within patients. Herein, we report a novel case of dapagliflozin-associated eu-DKA with unusual presentation. A 57-year-old female with a six-year history of type 2 diabetes mellitus (DM), for which metformin and dapagliflozin were prescribed, developed severe abdominal pain and signs of acute peritonitis without obvious hyperglycemia after distal pancreatectomy. Based on CT scan and laboratory studies, gastrointestinal tract perforation was suspected but was excluded during laparotomy. Severe metabolic acidosis and strong positive urine ketone indicated diabetic keto acidosis. The patient recovered after active therapy with an intravenous insulin infusion, antibiotics and correction of hypotension, electrolyte imbalance and acidosis. This case provides a new reference for clinicians and surgeons to be concerned with eu-DKA with severe abdominal pain as the main symptom.

## Introduction

Sodium glucose co-transporter-2 (SGLT2) inhibitors have become a popular therapeutic strategy in the management of hyperglycaemia in type 2 diabetes mellitus (DM) by inhibiting renal SGLT-2 receptors responsible for glucose reabsorption [1]. However, there has been a gradual increase in reports on diabetic keto acidosis (DKA) after administration of SGLT-2 inhibitors [2-6]. We herein report a rare case of euglycemic diabetic keto acidosis (eu-DKA) with severe abdominal pain as the main symptom, which was misdiagnosed as gastrointestinal perforation followed by unnecessary laparotomy.

## Case presentation

**Figure 1:**
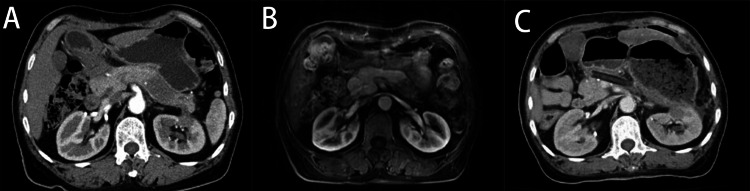
Abdominal computed tomography scan (A) and Magnetic resonance imaging (B) demonstrate a tumor in the tail of pancreas. Abdominal computed tomography (C) showed intra-abdominal free gas, peri-gastric exudation and possible discontinuity of gastric wall.

A 57-year-old female patient was admitted to our hospital for presenting with a six-month history of recurrent upper abdominal pain. The primary diagnosis was pancreatic tumor according to contrast-enhanced CT at another hospital. The patient had been taking appropriate oral medications for diabetes, hypertension and stroke for the past six years. Her prescribed oral hypoglycemic agents at the time of presentation were metformin (250mg twice per day) and dapagliflozin (10mg per day). The physical examination of the patient at admission showed no obvious abnormality. Serum tumor markers examination showed the following: carcinoembryonic protein, 10.6ng/ml (normal range <5ng/ml); carbohydrate antigen 19-9, 7205.6U/ml (normal range <37U/ml); carbohydrate antigen 242, 624.1U/ml (normal range <20U/ml). Urine test displayed positive of urine glucose and ketone. No obvious abnormality was detected in routine blood test and liver and renal function analysis. Magnetic resonance imaging (MRI) and CT showed a tumor in the tail of pancreas (Figure 1A, 1B). Laparoscopic distal pancreatectomy with splenectomy was performed. Histopathological analysis revealed moderately to poorly differentiated ductal adenocarcinoma at pancreatic tail without lymph node involvement (pT2N0M0). Continuous blood glucose monitoring after operation was performed, which was in normal range. The patient was gradually transitioned to semi-liquid diet and her oral hypoglycemic agents, metformin and dapagliflozin, were reinstituted.

However, on day eight after the operation, the patient developed abdominal pain accompanied by nausea. One day later, the patient’s abdominal pain was aggravated accompanied by tachypnea and increased heart rate (fluctuating between 110-130 beats per minute). Physical examination showed distended abdomen with mild tenderness and suspected rebound pain. Laboratory examination showed white blood cell (WBC), 16*109 /L with neutrophils occupying 87.8%. Other inflammatory parameters were also significantly elevated: procalcitonin (PCT) 2.66ng/ml (normal range <0.5ng/ml), C-reactive protein (CRP) 168.1mg/L (normal range <10.0mg/L). Drainage fluid biochemical examination showed an amylase content of 50600U/L indicating pancreatic fistula. Blood glucose test indicated moderately hyperglycemia (12.4mmol/L). Electrolyte analysis showed serum sodium 145.2mmol/L, potassium 5.26mmol/L. CT examination showed much more intra-abdominal free gas, peri-gastric exudation and possible discontinuity of gastric wall (Figure 1C). Considering the patient’s peritonitis sign, procedure-related gastric perforation was highly suspected. An emergency laparotomy was performed, and the digestive tract was carefully examined. The stomach was markedly dilated but without perforation. During operation, the patient displayed hypotension, which required vasoactive agents to maintain blood pressure. Arterial blood gas analysis showed a pH of 6.9, indicating severe acidosis. Myocardial enzymography and other tests were performed to rule out the possibility of myocardial infarction and pulmonary embolism.

After operation, the patient was sent to the intensive care unit (ICU) for further treatment. The status of hypotension and tachycardia (>130bpm) persisted initially. Arterial blood gas analysis still showed a picture of metabolic acidosis with an elevated anion gap (pH 7.09, CO2 56.7mmHg, HCO3- 17mmol/L, base excess（BE）-12.78mmol/L, anion gap 36.2mmol/L, blood glucose of 15mmol/L). Urinalysis examination showed strong positive of ketones, and serum lactate levels were normal (1.8 mmol/L). Electrolytes test showed significant hypernatremia (166.7mmol/L) and slightly hypokalemia (3.24mmol/L). The history of dapagliflozin medication and laboratory findings raised the concern for eu-DKA in the setting of SGLT-2 inhibitor use. The patient was therefore treated with an intravenous insulin infusion and fluid therapy, as well as antibiotics for preventing abdominal infection. Blood glucose and arterial blood gas were regularly monitored. Metabolic acidosis started improving and resolved over 9.5 hours (Table 1). The patient was discharged out of ICU on the fourth day after the second operation and no relapse of her DKA occurred for the remainder of her hospital stay.

**Table 1:** Laboratory data of the patient during hospitalization PaCO2: partial pressure of carbon dioxide, HCO3-: bicarbonate

parameter	Relevant range	Hospital Day 14 (18:30)	Hospital Day 14 (22:30)	Hospital Day 15 (4:00)	Hospital Day 15 (11:00)
PH	7.35-7.45	7.09	7.32	7.38	7.417
PaCO2(mmHg)	36.0-44.0	56.7	25.3	28.7	45.5
HCO3-(mmol/L)	22.0-26.0	17.0	12.7	16.4	28.8
Anion gap (mmol/L)	8.0-16.0	36.2	35.6	30.1	11.5
Blood glucose (mmol/L)	3.92-6.20	15	12.6	10.4	10.9
Sodium (mmol/L)	135.0-145.0	166.7	163.6	166.6	168.0
Potassium (mmol/L)	3.5-5.5	3.24	3.50	2.87	3.40
Serum lactate (mmol/L)	0.5-2.2	1.80	1.88	1.60	1.90
β-hydroxybutyric(mmol/L)	0.03-0.3			10.87	
glycated hemoglobin (%）	4.3-6.3			9.3	

## Discussion

Euglycemic ketoacidosis (euDKA) is a rare but fatal metabolic complication associated with SGLT2 inhibitor therapy [7], especially in the setting of extreme physical stress such as surgery, infection or acute medical illness. Despite a few cases that have been reported previously, our case is unique and highly challenging because: (1) acute abdominal pain with suspected peritonitis sign, actually caused by DKA, could be easily misdiagnosed as gastric perforation, due to elevated inflammatory markers, severe dilated gastric cavity, peri-stomach fluid collection, and much more free gas on CT images. Unnecessary laparotomy was performed which might be avoided if we know the pathogenesis of this disease; (2) the Hepato-Pancreato-Biliary (HPB) surgeons do not routinely prescribe these novel diabetic pharmaceuticals, and seldom know the possible side effects, especially eu-DKA. In classic DKA, patients often develop hyperglycemia due to accelerated glycogenolysis and an increase in glucogenesis, but patients on SGLT-2 inhibitors are generally normoglycemic or moderately hyperglycemic, a condition some physicians and surgeons may be unfamiliar with. Severe acidosis could be the clue for the diagnosis of DKA, which was further confirmed by positive urinary ketone. However, acidosis in this case could be reasonably explained by septic shock since the patient presented with hypotension and signs of infections. For these reasons as well as the rising incidence of diabetes and increased prescription of SGLT-2 inhibitors, HPB surgeons should be aware of these drug's potential adverse effects, especially acute abdominal pain and septic hypotension as main symptoms of eu-DKA, which might be quite confusing.

SGLT-2 inhibitors are new classical agents to overcome hyperglycemia by reducing the reabsorption of glucose in renal proximal tubules [8]. In addition, it can reduce body weight, blood pressure and the rate of cardiovascular death [9]. Compared with other SGLT-2 inhibitor-induced complications such as genital infections, urinary tract infections etc, eu-DKA is rare but life-threatening. By increasing the glucagon secretion, prompting hepatic ketogenesis, and inhibiting the reabsorption of glucose, SGLT-2 inhibitor keeps the patient euglycemic [10]. In our case, blood sugar level moderately elevated or in normal range after dapagliflozin readministration, which are far below the usual means of that in the traditional DKA. Due to no apparent hyperglycemia, delay in accurate diagnosis and inappropriate treatment exist in this case.

In previous reported cases, the main symptoms of eu-DKA are disturbance of consciousness, dyspnea, nausea, or vomiting [2-6]. However, no reports have described eu-DKA with severe abdominal pain (mimicking acute peritonitis) as the predominant symptom due to SGLT-2 inhibitors. In the present case, due to the history of surgery, abnormal inflammatory markers and imaging findings, the patient was misdiagnosed with procedure-related digestive tract perforation. The pathogenesis of abdominal pain caused by eu-DKA has the following possibilities: (1)the patient’s electrolyte disturbances, such as hypokalemia and hypocalcemia, lead to gastrointestinal smooth muscle dysfunction, which eventually lead to paralytic intestinal obstruction or acute gastric dilation resulting in abdominal pain; (2) Due to the high concentration of free fatty acid and high osmotic pressure, pancreatic microcirculation starts to break down, which lead to pancreatic swelling or exudation. Then the exudate irritates the peritoneum resulting in abdominal pain [11]; (3) Due to eu-DKA, the patient’s oxygen dissociation curve shifts to the left and oxygen release reduces. Hypoxia and a drop in pH of gastric juice induced by dehydration destroy the gastrointestinal mucosa and eventually cause acute gastritis [12]; (4) The accumulation of ketone bodies and acidic metabolites develop abdominal pain by irritating gastrointestinal smooth muscle. In our case, the intraoperative diagnosis of acute gastric dilatation and the amylase content of drainage fluid verify the above possibilities.

eu-DKA induced by SGLT-2 inhibitors shows the following physiological abnormalities: unexplained acidosis, hypernatremia, hypokalemia, hyperketonemia and uroketosis [2]. Unexplained acidosis might be the clue for the diagnosis of DKA, which could be further confirmed by positive urinary ketone. In our case, serum electrolytes disorder existed (hypernatremia 166.7umol/L and hypokalemia 3.24umol/L). However, ketone studies and blood gas analysis are not routinely examined in patients with diabetes after operation. Due to the confusing clinical manifestation, acidosis was not detected until at the time of re-operation. DKA was diagnosed when urinalysis indicated strong positive of ketones. Retrospectively, unnecessary laparotomy might have been avoided if we perform the ketone body examinations and blood analysis in advance.

## Conclusions

We report a case of a 57-year-old woman with type 2 diabetes who developed eu-DKA following pancreatic surgery. The findings of this case suggest that surgeries and infections can be potential predisposing factors for the development of SGLT-2 inhibitor-associated eu-DKA. When we encounter a diabetic patient presented with severe abdominal pain, a history of dapagliflozin and no significant hyperglycemia, we need to be aware of the possibility of eu-DKA. Arterial blood gas and ketone body examination should be investigated for prompt diagnosis and treatment. Misdiagnosis is quite common if we rely solely on symptoms and blood glucose.
